# An International Partnership of 12 Anatomy Departments – Improving Global Health through Internationalization of Medical Education

**DOI:** 10.5334/aogh.2665

**Published:** 2020-03-06

**Authors:** Anette Wu, Geoffroy P. J. C. Noël, Richard Wingate, Heike Kielstein, Takeshi Sakurai, Suvi Viranta-Kovanen, Chung-Liang Chien, Hannes Traxler, Jens Waschke, Franziska Vielmuth, Mandeep Gill Sagoo, Shuji Kitahara, Yojiro Kato, Kevin A. Keay, Jørgen Olsen, Paulette Bernd

**Affiliations:** 1 Department of Pathology and Cell Biology, Vagelos College of Physicians and Surgeons, Columbia University, New York, US; 2 Department of Anatomy and Cell Biology, McGill University, Montreal, Quebec, CA; 3 Department of Anatomy, King’s College London, London, UK; 4 Institute for Anatomy and Cell Biology, Medical Faculty, Martin Luther University Halle-Wittenberg, Halle (Saale), DE; 5 Medical Innovation Center of Kyoto University, Graduate School of Medicine, Kyoto, JP; 6 Department of Anatomy, University of Helsinki, Helsinki, FI; 7 Department of Anatomy and Cell Biology, College of Medicine, National Taiwan University, Taipei, TW; 8 Center for Anatomy and Cell Biology, Medical University of Vienna, Vienna, AT; 9 Institute of Anatomy, Faculty of Medicine, Ludwig Maximilians University, Munich, DE; 10 Department of Anatomy, Tokyo Women’s Medical University, Tokyo, JP; 11 Department of Surgery, Kidney Center, Tokyo Women’s Medical University, Tokyo, JP; 12 Discipline of Anatomy and Histology, University of Sydney, Sydney, AU; 13 Department of Cellular and Molecular Medicine, University of Copenhagen, Copenhagen, DK

## Abstract

**Background:**

At a time of global interconnectedness, the internationalization of medical education has become important. Anatomy as an academic discipline, with its close connections to the basic sciences and to medical education, can easily be connected with global health and internationalization of medical education. Here the authors present an international program based on a partnership between twelve anatomy departments in ten countries, on four continents. Details of a proposed plan for the future direction of the program are also discussed.

**Objective:**

The aim is to improve global healthcare by preparing future global healthcare leaders via early international networking, international collaboration and exchange, intercultural experience, and connecting two seemingly distant academic disciplines – anatomy and global health – via internationalization of medical education.

**Methods:**

Based in the anatomy course, the program involved early international collaboration between preclinical medical and dental students. The program provided a stepwise progression for learning about healthcare and intercultural topics beyond pure anatomy education – starting with virtual small groups of international students, who subsequently presented their work to a larger international audience during group videoconferences. The above progressed to in-person visits for research internships in the basic sciences within industrialized countries.

**Findings:**

Students appreciated the international and intercultural interaction, learned about areas outside the scope of anatomy (e.g., differences in healthcare education and delivery systems, Public and Global Health challenges, health ethics, and cultural enrichment), and valued the exchange travel for basic sciences research internships and cultural experience.

**Conclusions:**

This unique collaboration of international anatomy departments can represent a new role for the medical anatomy course beyond pure anatomy teaching – involving areas of global health and internationalization of medical education – and could mark a new era of international collaboration among anatomists.

## Background

At a time of global interconnectedness, internationalization of medical education (IoME) has become an important part of medical education.

Internationalization in higher education is “the intentional process of integrating an international, intercultural, or global dimension into the purpose, functions, and delivery of post-secondary education, in order to enhance the quality of education and research for all students and staff and to make a meaningful contribution to society [1].”

Internationalization of medical education (IoME) is a term used in studies of the education literature [2,3,4,5,6], and has not been the focus of major research. IoME can include exposure to both developing and developed countries’ health contexts and issues, which is in line with the definition of global health (GH) [7].

There are several models that have been described as the rationale for pursuing IoME. Hanson (2015) describes a market model, a liberal model, and a social transformation model [8,9]. In the market model, institutions and countries aim to strengthen their position internationally to achieve or maintain a competitive edge within the global market. The liberal model promotes international collaboration and intercultural understanding. In the social transformation model, an analysis of the social impacts of globalization, with resulting inequalities, marginalization of people, and interdependencies, is the driving force to address social injustice.

It is important for healthcare practitioners and leaders to practice medicine with a global mindset, and internationalization can enhance learning about key issues that are specific to health education worldwide [10]. Because IoME can enhance students’ understanding of social, cultural, and ethical differences, it can prepare future physicians to practice with a global frame of reference and a better understanding and awareness of cultural differences [10], thus ultimately improving GH for all people worldwide.

IoME can have different formats involving various stakeholders and dimensions of medical education. It can address student issues and experiences, faculty related topics, and/or the curriculum itself [11]. Furthermore, internationalization elements can be seen at the level of institutions, governments, or policies (e.g., in the form of university consortia, and international governmental partnerships).

At the student level, IoME can involve didactic lectures in the classroom, peer-to-peer connections, outbound mobility activities for international student travel [12], and inbound mobility dimensions by increasing the proportion of international students.

In the US, it appears that the social transformation model is the predominant reason for IoME. Often, reports on IoME are part of a GH initiative and as such most reports revolve around placements of clinical students and students’ clinical or research experiences in low- and middle-income countries (LMIC) [13].

In Europe, international exposure has a long tradition (i.e., via ERASMUS, IFMSA, and DAAD) and institutional or governmentally supported programs are common [14,15,16]. These programs were often established in line with the liberal model following efforts to support integration and cultural understanding in the post-World War II era [17]. Growth can also be seen as a major driver for IoME in certain countries [18,19].

Internationalization in medicine as an educational field does not seem to have standardized curricula or agreed-upon learning objectives [12,20,21,22,23,24], has different home departments within medical schools, and is often embedded in programs within schools of public health and GH [25].

### Anatomy collaborations and internationalization

Anatomy is present in all health sciences curricula. Although international collaborations among anatomy departments can be advantageous, the existing cases have not been extensively reported recently, with even fewer reports involving international anatomy collaboration in the LMIC [2,26,27,28].

Anatomy as an academic discipline, with its close connections to the basic sciences and to student education, can easily be connected with GH and IoME. In his keynote address at the meeting of the American Association for Anatomy (AAA) in San Diego (2018), Dr. Jeffrey Murray, Deputy Director of the Bill and Melinda Gates Foundation, encouraged the involvement of anatomists in international work and GH [29].

This article describes the efforts of twelve anatomy departments in ten countries, on four continents, who collaborated in a unique program to promote IoME, with the ultimate goal of improving global healthcare. While the program was purposefully anchored in the anatomy courses, the content for student experiences expanded beyond anatomy and involved areas of public and GH. The authors have previously reported on a limited pilot study regarding this novel approach and are now presenting the full program along with updated results of student questionnaires [30].

## Objective

The goal of the program was not to internationalize anatomy teaching or the anatomy course content. The anatomy courses rather served as a vehicle and binding element to help improve global healthcare by preparing future medical and dental leaders via early international networking, international collaboration and exchange, and intercultural experience.

Anatomy was chosen as a commonality for student networking because this subject field is generally taught at an early stage and is represented in all medical and dental school curricula around the world. In addition, the topic of body donation and associated concerns about the topic of death are areas that are shared by young students in this phase of learning.

The program provided a structure for students to exchange knowledge and learn about other medical education and healthcare systems, differences in health law and ethics, public health challenges, and to be introduced to basic sciences research, along with immersion in an academic life abroad and intercultural exchange. To the authors, these elements seemed important to include in the preparation of future global healthcare leaders, and to help them to approach their future practice with a global frame of mind. By preparing global healthcare leaders, the program hopes to improve global healthcare.

The results of this publication focused on short-term outcomes regarding the learning of relevant competencies via the program. Although global competencies in medical education are not clearly agreed upon in the literature [12,23], competencies here included learning about international health topics and issues, working in international collaborative groups, and enhancing cultural competencies via cultural presentations, international public speaking in large group conferences, and experiences obtained from international student internships (see below).

## Methods

### Partners

Led by Columbia University, this program was a collaboration of twelve universities in ten countries, on four continents (Table 1). In the first phase, only partners from industrialized countries were included. The program was situated in the anatomy course and included very young and inexperienced students. A significant number of the students were in their late teenage years or early twenties; some of the students had never travelled outside of their home countries. Purposefully, partners were selected that were similar but culturally diverse enough for these young students to appreciate the differences without being overwhelmed. There are plans for more diverse student groups in the program’s second phase.

**Table 1:** List of partner schools and corresponding student numbers, including inbound and outbound student travels.

Partner Universities	Student numbers for small group collaboration	Outbound Student numbers	Inbound student numbers

Medical University of Vienna, Vienna, Austria	12	6	5
The University of Sydney, Sydney, Australia	35	9	2
McGill University, Montreal, Canada	23	0	5
University of Copenhagen, Copenhagen, Denmark	3	1	1
University of Helsinki, Helsinki, Finland	10	9	1
Ludwig Maximilians University, Munich, Germany	8	5	3
Martin Luther University, Halle, Germany	15	3	4
Kyoto University, Kyoto, Japan	11	1	4
Tokyo Women’s Medical University, Tokyo, Japan	9	4	1
National Taiwan University, Taipei, Taiwan	8	6	3
King’s College, London, United Kingdom	24	2	4
Columbia University, New York, United States of America	36	18	34
**Total**	194		

Partner schools in phase 1 were carefully evaluated for a number of inclusion criteria (e.g., safety of the country for student travel, availability of quality research opportunities, strong history regarding medical education, and a rich cultural history). The vetting process, while subjective, included an initial online search for leading schools with strong scientific research opportunities, as well as recommendations from professional scientific colleagues. In addition, pre-selected partners that were already collaborating with Columbia University were included. Initial connections were made via their respective anatomy departments, and through anatomical professional societies. Twelve universities participated.

The second phase includes experience with LMIC, is in the pilot stage, and not part of this report; its format is addressed at the end of the program structure (see below, part 2).

### Program structure

The program was a voluntary program and accessible to all preclinical medical and dental students. The focus was on very junior students. Therefore, a stepwise progression was provided, starting with bonding in small and subsequently larger video groups, proceeding to in-person visits to industrialized countries in phase 1, before moving on to in-person visits to LMIC in phase 2 (pilot stage). All participating students were encouraged to remain in contact with their international peers for future life-long professional networking.

The preclinical program in phase 1 currently enrolls approximately 200 students per year (see Table 1 for distribution by countries). Phase 1 was operating in its sixth year at Columbia University at the time of this publication. Short-term results of the success of the program in phase 1 were evaluated by limited qualitative data (i.e. the students’ perceptions of the program).

#### Local liaisons

Because of the connection via the anatomy departments, each school had one dedicated international anatomy faculty member serving as a contact. Although the program was initiated by Columbia University, all schools and faculty members were considered equal to one another. Regular faculty meetings online and email communication with all partners ensured a constant flow of communication and transparency.

Every year, each school selected up to two volunteer national student leaders (NSL) who led the student cohort from their respective schools and represented their school and country at the videoconferences (see program details below). The NSL formed their own sub-leadership groups and worked closely with the coordinating entities at Columbia, thus representing a second level of communication in this multi-member collaborative partnership.

### Part 1 – Industrialized countries

#### Small group work

The purpose of the small group work was to give students a framework to practice international teamwork, develop an understanding of others’ customs, and to familiarize themselves with differences in culture and work ethics.

Small groups typically consisted of three to five students from three countries (at least one each from North America, Europe, and Asia/Australia). Beginning in the fall, students met online for one semester to work in small groups during several structured sessions. Session topics covered the differences in the anatomy courses, body donation policies, healthcare education and delivery systems, health law and ethics, and public health.

The program content goal deliberately went beyond anatomy and its related topics. Only one of the small group sessions was dedicated to discussions related to anatomy and the practice of body donation. The purpose of this first session/exercise was to serve as an “ice-breaker” and to reflect on subjects that were familiar to all participants (i.e., anatomy, body donation, working with the deceased). In this first session, the students were asked to compare the format of their anatomy courses, followed by reflection on the topic of body donation in self-reflection and as an international group. Body donation processes can vary between countries, even between schools in one country (e.g., some schools accept direct donations from families while others have a centralized system; some schools pay the donors). Because body donation is handled so differently, it serves as a tool to raise awareness about cultural differences. A list of suggested discussion topics for the remaining sessions was initially developed by faculty and further expanded upon over the years per students’ requests (Table 2).

**Table 2:** Selection of discussion topics beyond anatomy. Topics were expanded every year.

Discussion Topics	Selection of Topic

Healthcare Education	Differences in medical school curriculum
	Differences in postgraduate and residency training
	Differences in tuition
	Differences in salaries
Healthcare Delivery Systems	Differences in healthcare delivery systems
	Differences in health insurance systems
	Differences in remuneration and fees
	Differences in hospital systems and general medical practice
	Shortage of healthcare workers
Public Health Challenges	Aging
	Obesity
	Epidemics (Tuberculosis, Ebola, HIV/Aids)
	Addictions
	Mental Health
	Environmental Health/Climate and Health
	Healthcare access and health equity (social justice)
	Socio medical sciences
	Immigrant health
Health Ethics and Law	Abortion
	Euthanasia (Aid-in-dying)
	Organ donation law
	Stem cell and embryonic research
	Contraception
	Infertility treatment law (egg donation.
	genetic testing, surrogacy)

Toward the end of the small group sessions, the students worked with their peers on a short collaborative small group paper covering one topic from the aforementioned list, creating a video and slide presentation together. The reasons for producing the paper and presentation were to strengthen their collaborative efforts, to communicate efficiently, to work across different time zones, to exercise professionalism and tolerance of others in an international setting, and to practice international public speaking at the videoconferences.

#### International student conferences

**Figure 1:**
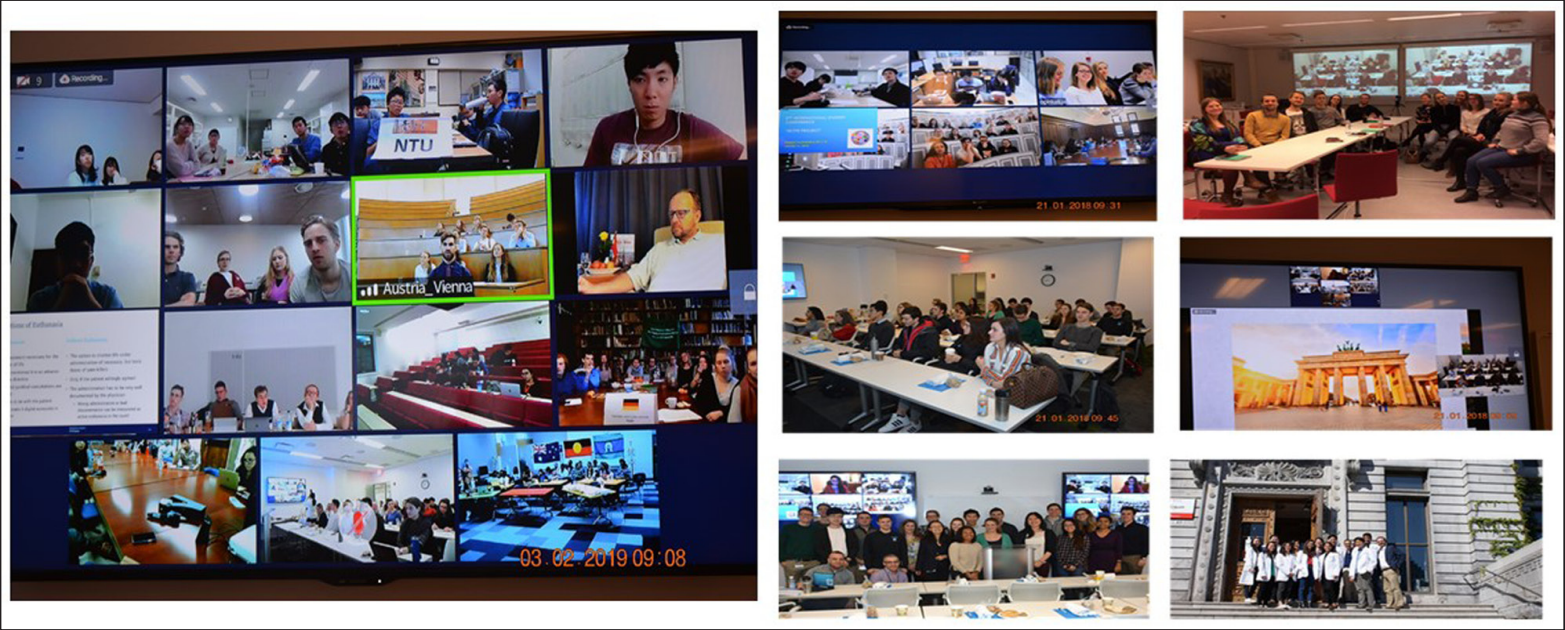
Online student conference.

At the end of the semester the students presented their collaborative small group work to the entire cohort at two large international virtual conferences, with all students and faculty participating (Figure 1).

The groups of students from each country met in their respective lecture halls, libraries, or AV rooms, and connected via an online videoconferencing program (Zoom)©. NSL from each country served as moderators for the conferences, with minimal faculty involvement – although faculty was present with their groups online.

The conferences were divided into three blocks. Block 1 included a cultural presentation by the respective NSL. The presentation covered various characteristics (e.g., history, geography, art, architecture, customs, stereotypes, food, etc.) of each country and region. The purpose of the cultural exchange was to introduce different cultures and customs to these junior students and to inspire them to learn about a different country beyond the perspective of healthcare.

Block 2 included selected international group presentations on the topics the students had previously chosen for their collaborative papers. This exercise was practice for international public speaking and listening, developing an appreciation of non-English speaking students, and for non-native speakers to practice their fluency in English.

Block 3 included an introduction of the students that had expressed an interest in international exchange. Students introduced each other and paired up as “buddies”. This portion was meant for virtual international networking and was the only time when all students met together online.

The two international online conferences ended in early spring, completing the semester of virtual exchanges.

#### Student mobility

Subsequently, in the summer following the conferences the students travelled to the partner countries in order to perform short-term research in basic science laboratories (from 1–2 months up to 12 months). In addition, they socialized with their peers in the host country. This portion of the program was introduced to deepen collegial friendships, help students to immerse themselves in an academic life in another country, and for them to acquire research skills in the basic sciences.

Research laboratories were selected based on the students’ preferences for an area of research (e.g., immunology) and institution, subject to availability in the host laboratories of the partner countries (Table 3).

**Table 3:** Basic sciences research areas for travel. Research laboratories were assigned per students’ preferences and determined by the availability of the host university.

Partner Universities	Research Area offered

Medical University of Vienna, Vienna, Austria	Tissue Engineering
The University of Sydney, Sydney, Australia	Stem Cell
McGill University, Montreal, Canada	Immunology, Neuroscience
University of Copenhagen, Copenhagen, Denmark	Diabetes
University of Helsinki, Helsinki, Finland	Lipid Physiology
Ludwig Maximilians University, Munich, Germany	ENT, Transplantation Immunology, Neuroscience
Martin Luther University, Halle, Germany	Immunology
Kyoto University, Kyoto, Japan	Neuroscience
Tokyo Women’s Medical University, Tokyo, Japan	Neuroscience
National Taiwan University, Taipei, Taiwan	Cancer Immunology, Bioengineering, Public Health,
King’s College, London, United Kingdom	Neuroscience
Columbia University, New York, United States of America	Immunology, Pathology, Surgery, Gynecology, Public Health/Epidemiology, Neuroscience, Precision Medicine, Tissue Engineering

The anatomy faculty in each country assisted with laboratory placement, and supported grant applications. Funding sources varied (i.e., departmental, school, or university scholarships and stipends, governmental, GH program), and students also self-funded their trips. Peers helped each other with travel logistics, accommodations, leisure activities, and facilitated immersion into academic life in the host countries.

Student visits were divided (by countries) into several travel groups, to ensure that not all students travelled at the same time and that participating students were available for hosting the incoming students in each country. Incoming students experienced a rich social program hosted by peers and faculty (e.g., cultural activities, walking tours, picnics, private parties, etc.). This allowed for mingling with their peers during their stay abroad, outside of their time involved in a research project. A constant flow of new incoming international short-term students internationalized the host campuses.

#### Evaluation

Program evaluation can be separated into short-term and long-term results. Short-term evaluations had two purposes – evaluate the program itself (i.e., via feedback about what students liked and how the program could be improved), and assess student learning (i.e., to measure what they felt that they had learned). In this program, qualitative data included students’ perceptions of what they had learned.

Questionnaires (Qualtrics)© were sent to the students, after both the virtual and the travel portions of the program, to evaluate the success of the endeavor. Questions included closed and open questions and Likert scales. Success was measured using students’ perceptions of their satisfaction with the above experiences, their knowledge about covered topics and basic sciences research, and their fundamental understanding about other cultures and healthcare systems. Questionnaires in Qualtrics were developed in collaboration with the Center for Education Research and Evaluation (CERE) at Columbia University and were approved by the IRB at Columbia University (#AAA0003715) and at McGill University (#A07-E54-17B).

Long-term evaluation to measure what effect the program had on the students’ careers and lives, and subsequently on global healthcare, could not be evaluated at this stage.

#### Software and computer programs

Questionnaires were collected using Qualtrics. Analysis of data was performed using NVivo12© and Excel©. Online software used for the small group discussions included Facetime and Skype. The large group conferences were conducted via a university-licensed Zoom account.

Since the students only worked with peers from two other countries, they connected and socialized with the larger group via closed social media sites (i.e., Facebook©, Instagram©, and Twitter©).

### Part 2 (future direction)

A recently initiated, second phase of the program, is in the planning stage and represents a progression from industrialized to LMIC. It involves a scholarly project conducted during the senior clinical years, available to prior program participants. Students who participated in part 1 are offered online international small group projects addressing global and public health issues in the LMIC, which culminate in clinical observership placements in the LMIC (Table 4). Components and results will be shared at a later time.

**Table 4:**
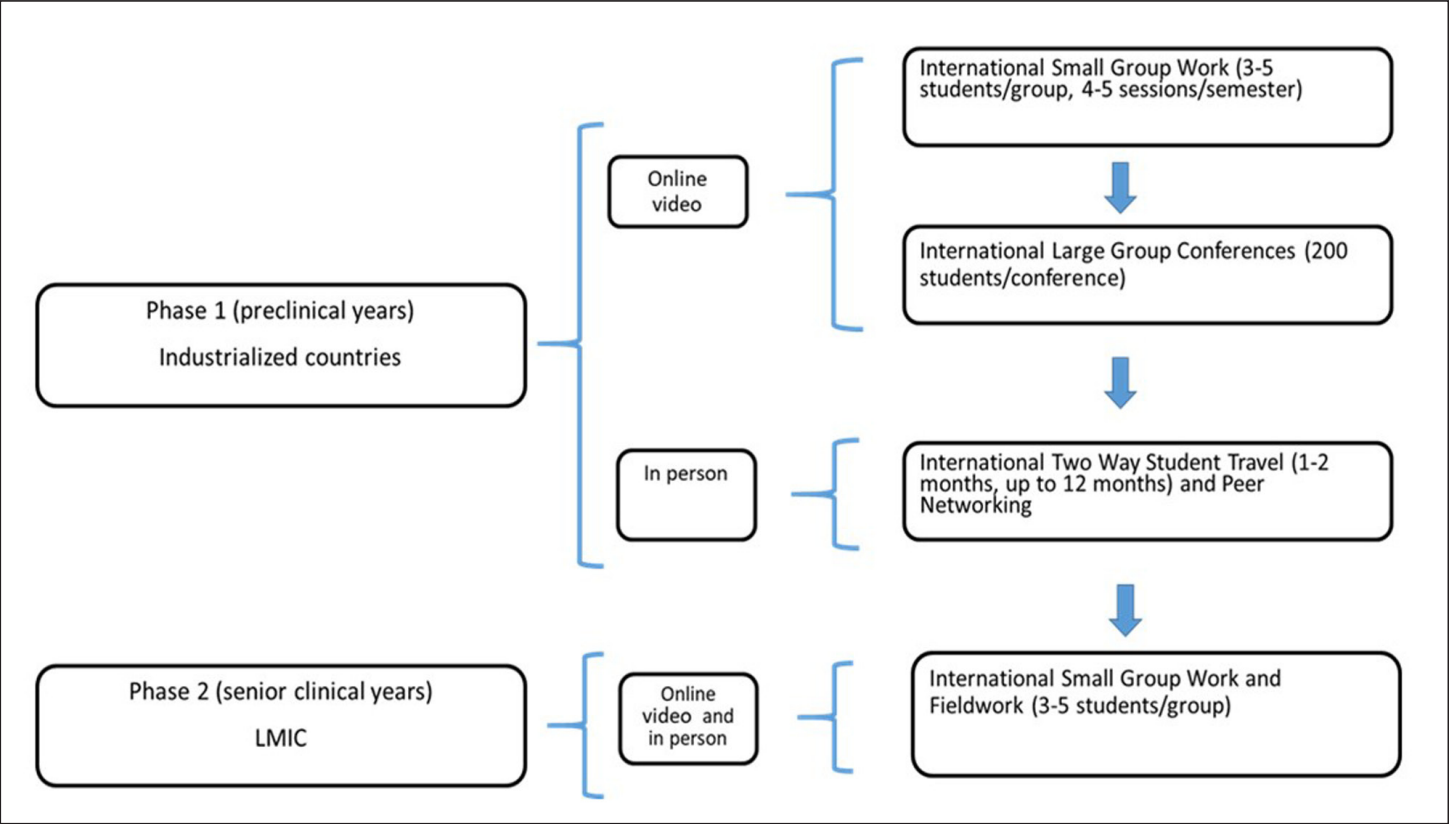
Overview of the format of the program structure (including pilot phase 2). A stepwise format eased students into international experiences.

## Findings

Results represent the academic year of 2018/19.

### Demographics

Of 194 participating students, 40% were male and 60% were female. Twenty-eight percent of the students were under the age of 20, and 72% were under 25 years old.

Ninety-two students responded to the program questionnaires (46%), and 99 (49%) replied to a separate questionnaire on the topic of body donation. Of those responding to the latter questionnaire, 19% were under the age of 20, and 63% were under 25 years old.

The low return rate was expected because the program was a voluntary program.

### Analysis of Program Format

Small group sessions. The majority of students enjoyed the small group interaction. On a Likert scale of from 1 to 10, the mean ranking reported was 8.46 (data not shown). The appropriateness of the interactions based in the anatomy course, the selection of discussion topics, and the desire to keep in contact with their peers were positively rated and consistent with previous years [30].

Conferences. 80% of the students liked the conferences (data not shown). When asked to rate their prior experience in international speaking engagements on a scale of from 1 to 10 the mean rating was 4.23, indicating that students did not feel that they had much experience (although some students did have past involvement – data not shown).

**Figure 2:**
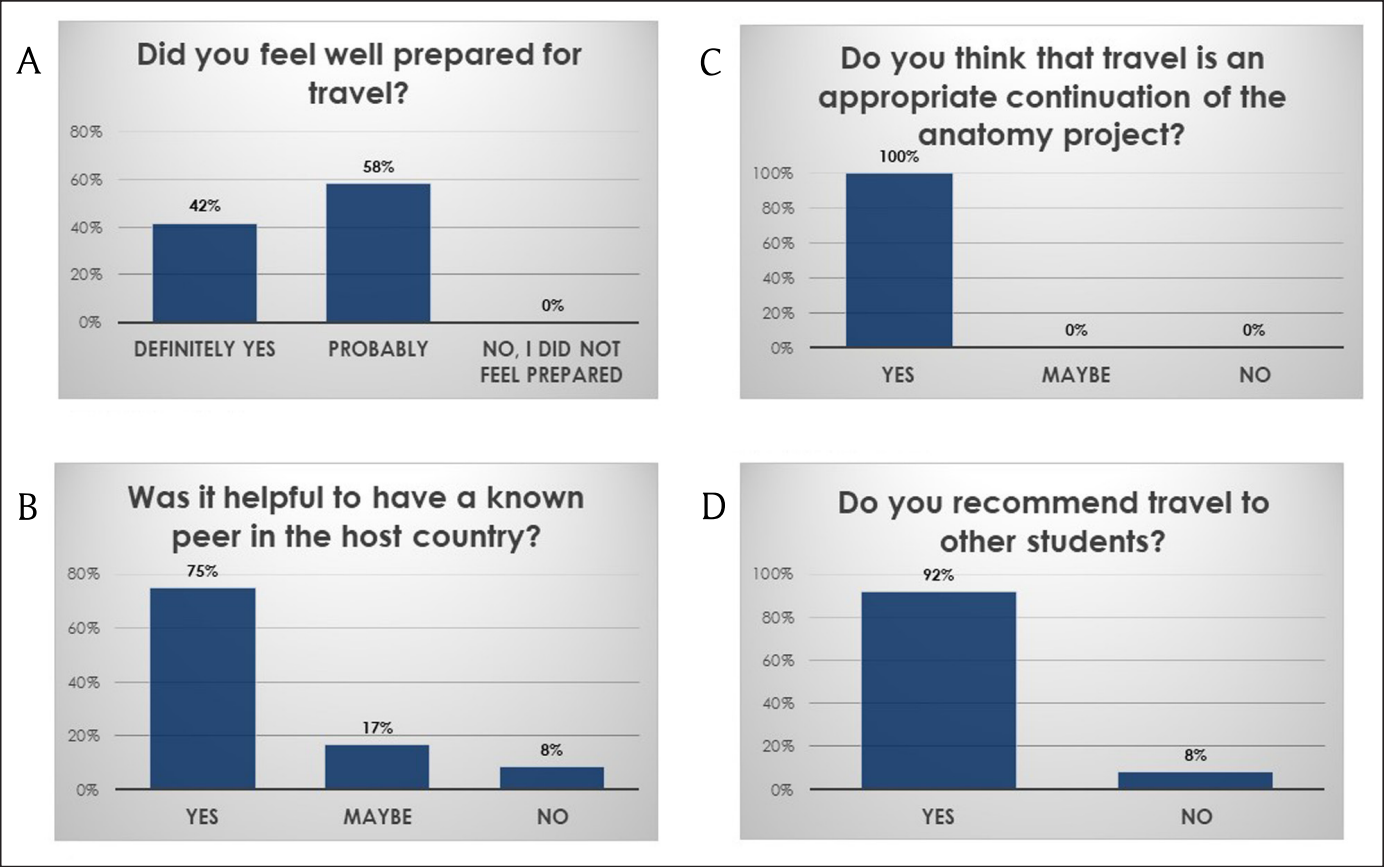
Student travels results (n = 12).

Student mobility. In 2018/2019 about 80 students travelled (Table 1). However, only a limited number of students travelled before 2018, and most did not participate in responding to the post-travel questionnaire. So, very preliminary post-travel data are currently available (n = 12; Figure 2A–D). Students rated their international travel experience, with a mean ranking of 9 overall (on a Likert scale of from 1–10 – data not shown).

### Analysis of Program Content

**Figure 3:**
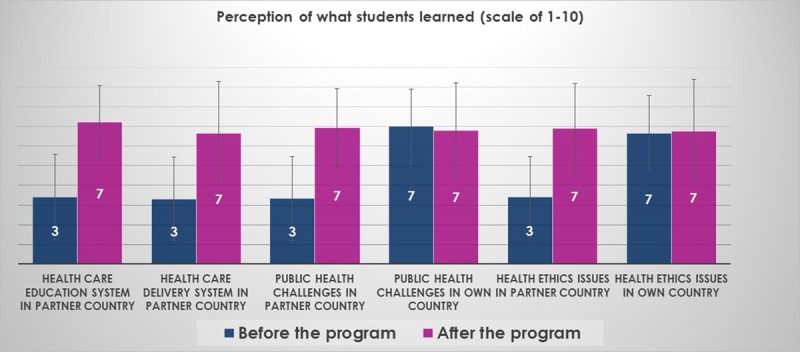
The majority of students learned about the healthcare education, healthcare delivery, health ethics, and public health challenges in the partner countries but did not feel that they learned more about these topics in their home countries.

**Figure 4:**
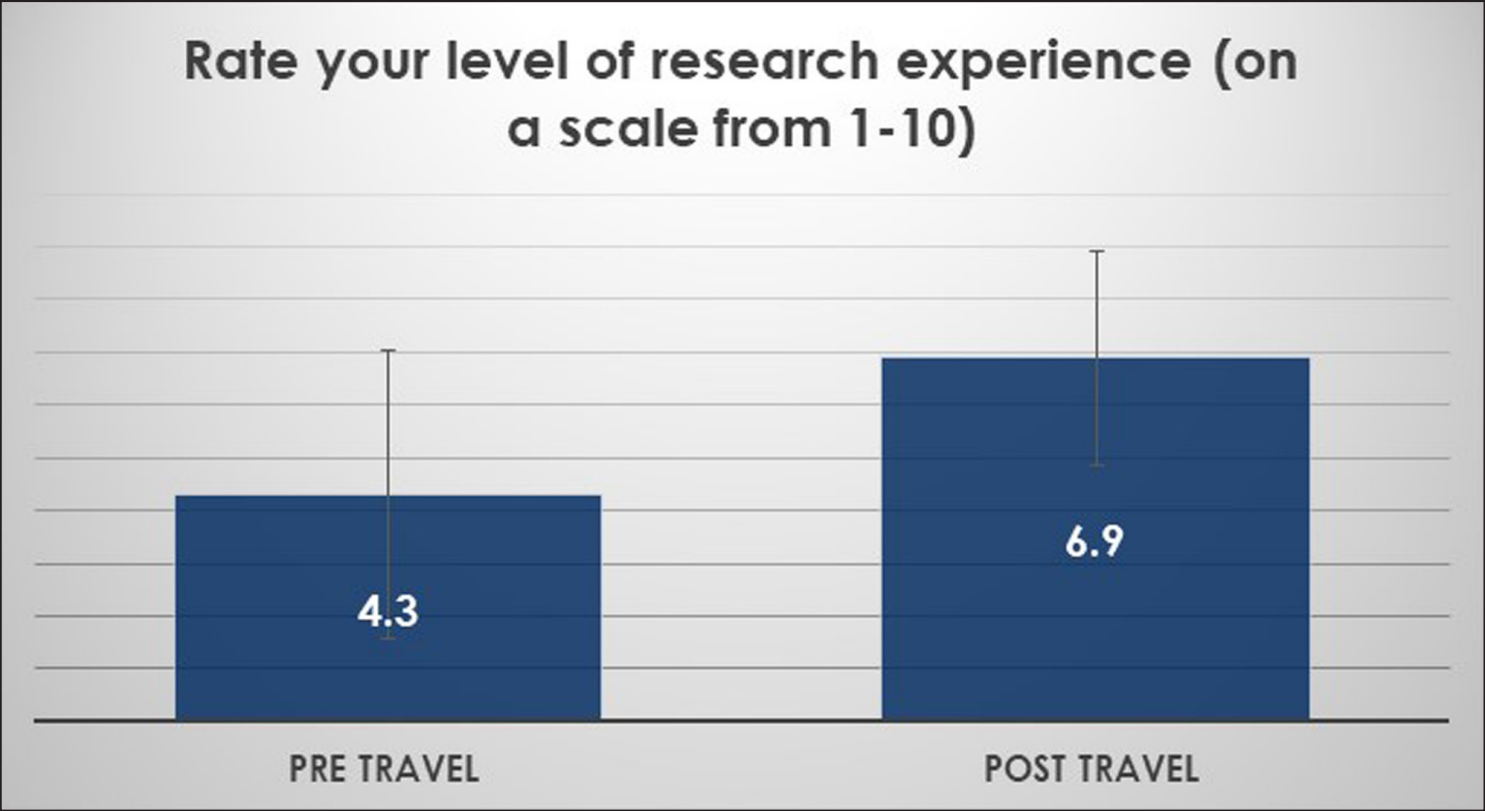
Research abroad experience. Students felt they improved their research skills. The experience abroad was meant to improve research skills but not to improve above the level of what students would have experienced in their home countries. No control was available.

**Figure 5:**
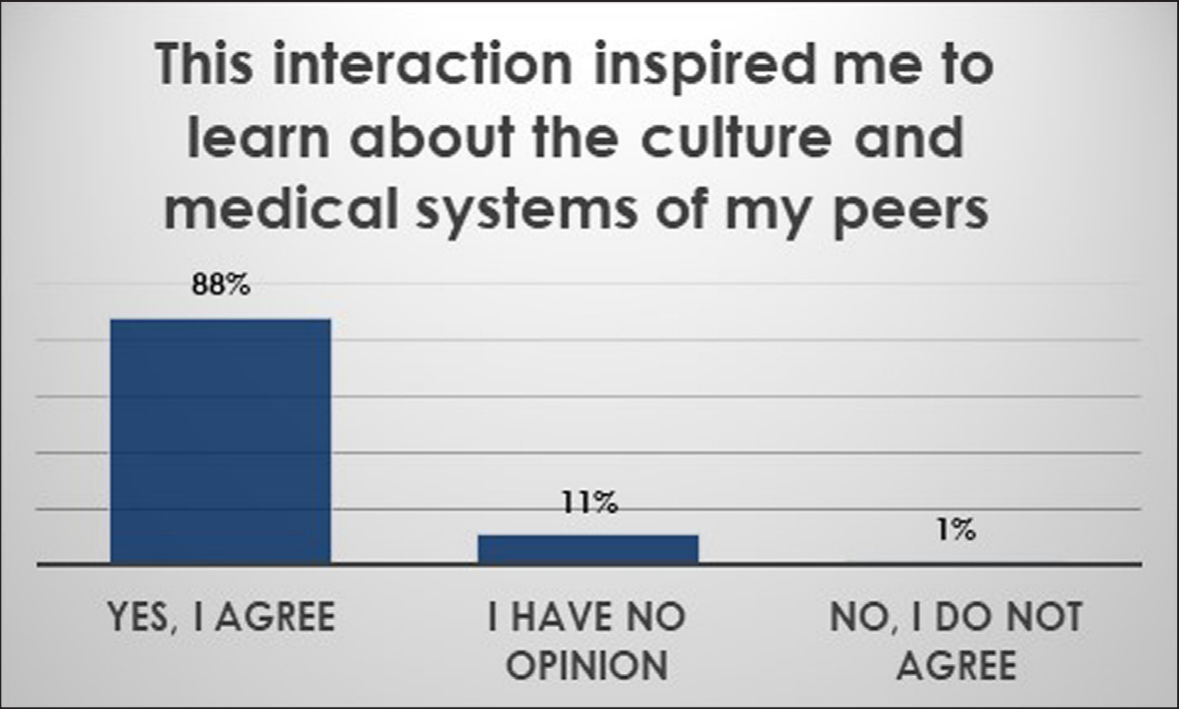
Students felt inspired to learn more about the other countries’ culture and medical systems.

**Figure 6:**
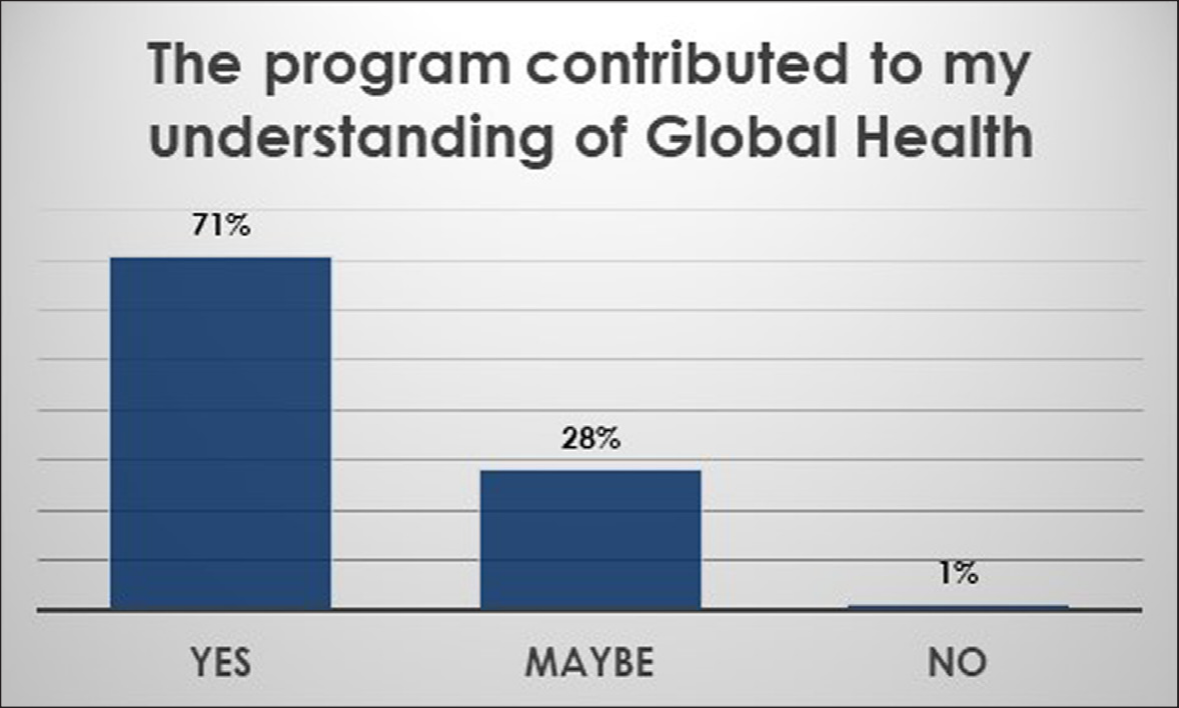
The majority of students felt that the program contributed to their understanding of Global Health.

Beyond anatomy topics. Overall, students felt that they had learned about healthcare education, healthcare delivery, public health, and health ethics in the partner countries during the small group sessions and large group conferences (Figure 3). In addition, basic sciences skills improved (Figure 4). Thematic analysis of open-ended questionnaires using NVivo software showed the themes that the students wrote about in regard to their learning experiences included a wide range of topics (Table 5) and inspired them to learn more (Figure 5). The students felt that the program contributed to their understanding of GH (Figure 6).

**Table 5:** Theme analysis of what students learned. Students perceived learning about a variety of topics beyond anatomy related topics.

Themes	Students’ responses

Medical education systems	“US medical education is so expensive. There is a really big lack of female medical professionals in Japan.”
Healthcare delivery systems	“Although countries seem to differ in healthcare systems, through our discussions we realized that some parts of delivery were quite similar and could lead to future collaboration!”
Health insurance systems	“I learned all about the Bismarck model of health insurance and how Japan and Germany handle their healthcare expenses as compared to the U.S.”
Health law and ethics	“Each country has the same ethical thoughts about abortion.”
Public Health challenges	“Challenges are quite similar all over the world.”
Anatomy course and related topics	“You have to pay to donate your body in Germany.”
Politics and health	“Health problems contain political issues”
Cultural differences	“Cultural shock!”

**Figure 7:**
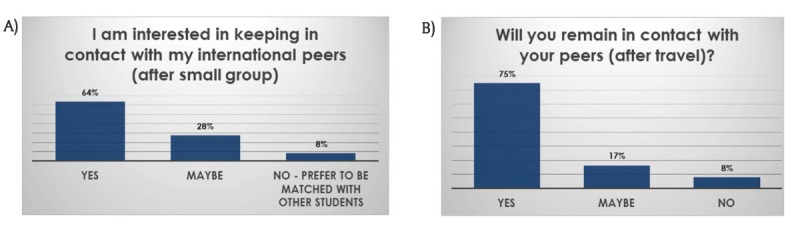
**A)** The students felt connected and wanted to remain in contact with each other after the small group work. **B)** After traveling to the partner countries the students were motivated to remain in contact with their peers (over 90%;). The percentage of interested students increased after their travels.

**Figure 8:**
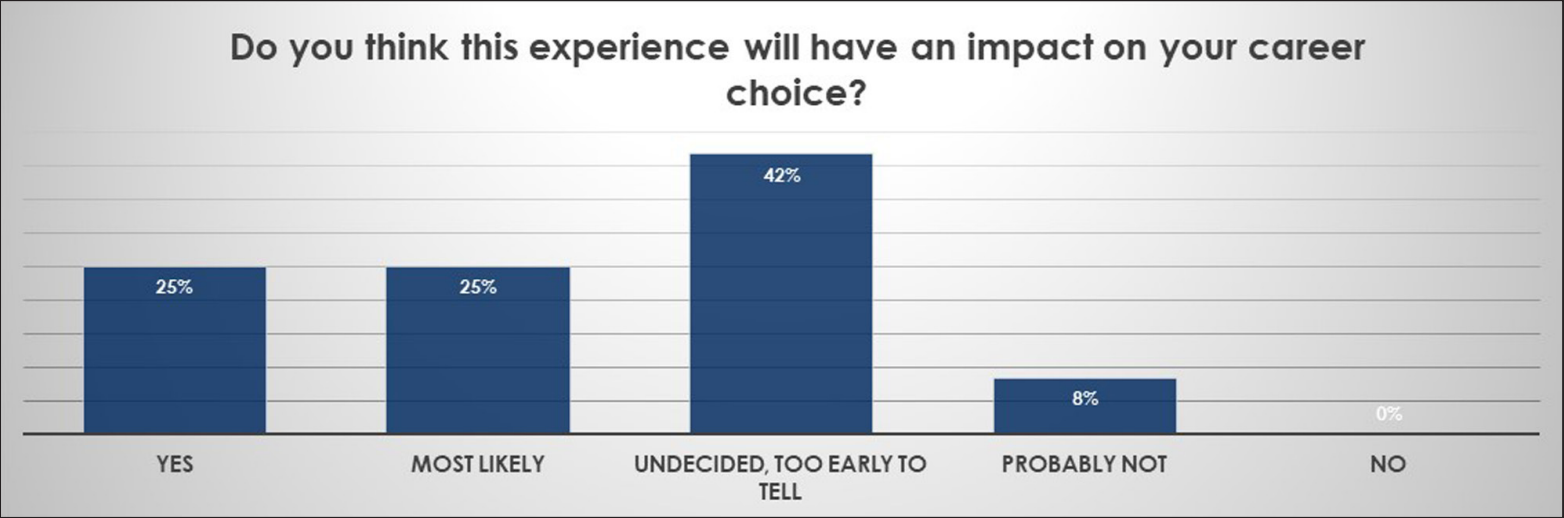
Impact on future career choices. The majority of students felt this interaction might have an impact on their career choices.

Networking. After their travel (Figure 7B), more students (compared to after the online portion of the program, Figure 7A) responded that they will remain in contact with their peers, indicating the importance of in-person networking (Figures 7A and 7B). The majority of students (82%) wished to continue the program into the clinical years (data not shown) and felt that the experience might have an impact on their career choice (Figure 8).

**Figure 9:**
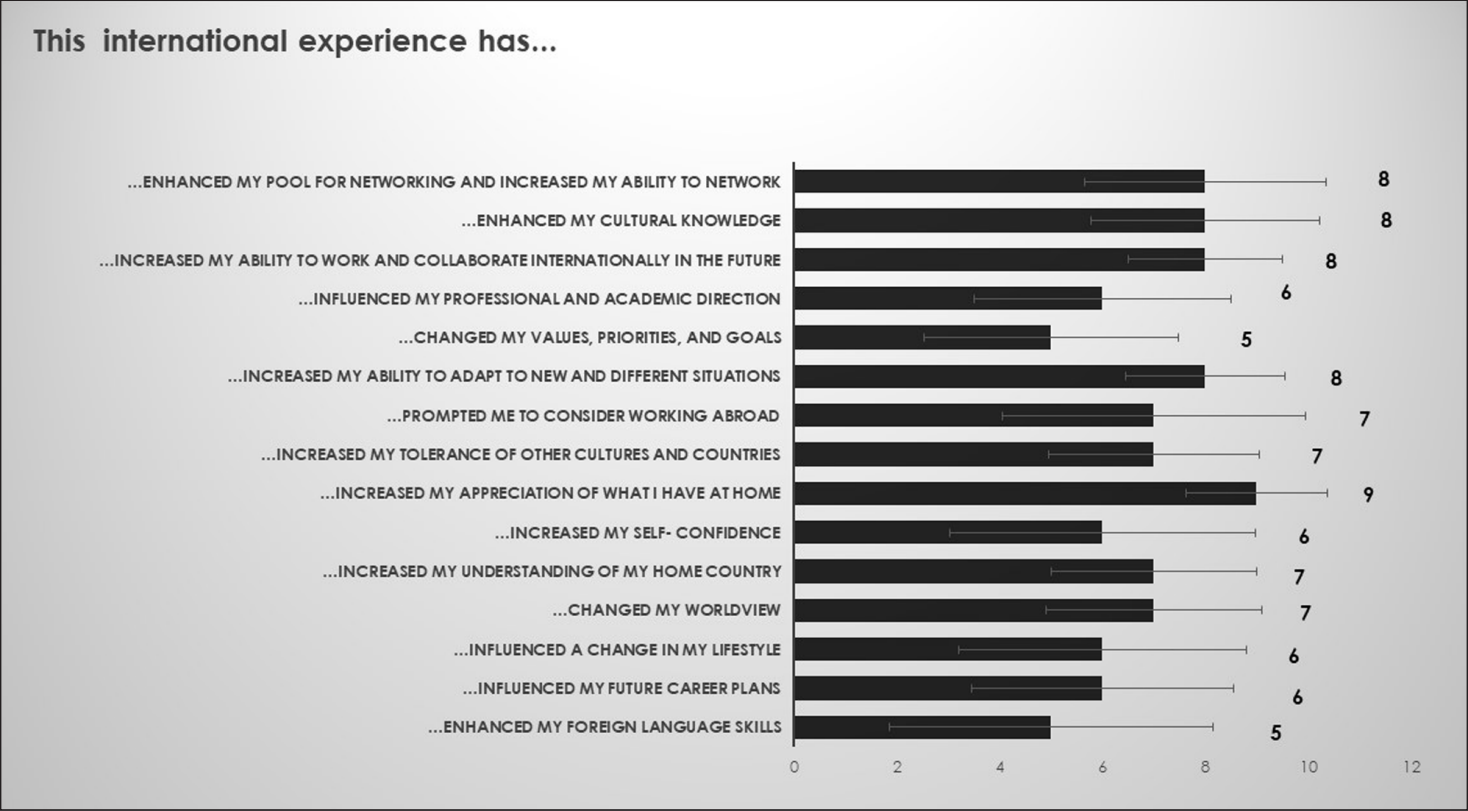
Responses from student questionnaires on overall learning and “hidden curriculum” (on a scale from 1 to 10).

“The hidden curriculum”. To measure skills that are not linked to the immediate program goals, some questions covered what additional skills the students thought that they gained. Students felt that they gained experience/knowledge in a variety of areas of “hidden” learning objectives – i.e., tolerance, appreciation, self- confidence, change in world-view, and influence on lifestyle changes (Figure 9). Students learned from each other about differences in body donation processes (data not shown). Results from their reflective written pieces on the topic of body donation are complex and beyond the scope of the description of an international program. Data are currently being analyzed (manuscript in preparation).

In summary, this program offered students insight into other international healthcare education systems and healthcare delivery systems and sensitized the students to intercultural differences. Also, they learned about international public health challenges, other countries’ health ethics and health laws, international ethics on body donation, and obtained a global picture of healthcare. In addition, the program taught the students about differences in body donation processes in different countries, and helped them to reflect on this sensitive topic.

## Discussion

This report adds to the body of available literature on IoME with the format, content, and results of a new international student exchange program that was built upon a unique partnership of multiple international anatomy departments. Anatomy as an educational discipline was utilized as a binding element for the international work.

The program included formal elements of IoME such as university partnerships, student education, and student mobility. Program content encompassed elements of the liberal, social justice, and competitive models to promote future international collaborative problem solving in healthcare, with the ultimate goal of improving GH.

### Format of the Program

#### Stepwise introduction

Very few published programs build on a trajectory of international experiences and involve both industrialized and LMIC countries [31]. Unlike others, this program involved a stepwise and sequential introduction of international experiences.

International experiences, particularly in the US, frequently aim to support the social justice notion of IoME, and historically often involved humanitarian work in the LMIC as part of GH programs [12,23]. The authors suggest that these goals need to be revisited at a time of espoused global social equality and accountability and should be more inclusive of all aspects of IoME. In contrast, European and Asian schools and their exchange programs tend to have a broader scope, with student mobility including industrialized countries, most often the Anglo-Saxon countries [14] – to support competitiveness and collaboration.

Often, there is little introduction to acquaint young students with international differences, although pre-departure training is considered important and offered in recent years [21,32]. The students in this program felt that they were well prepared in many areas for travel, such as culture, emotional wellness/culture shock, knowledge, and safety [32].

#### Early internationalization

Reports on early internationalization efforts for junior medical students are limited [3,6,33,34]. In this program, the majority of the participating students were still in the formative phase of their lives. The authors assert that early international connections have a higher chance for long-term sustainability of the newly formed collegial friendships. To date, the program has not been in existence long enough to have longitudinal results. It was encouraging to learn that the majority of students planned to remain in contact with each other, which indicates potential long-term sustainability. Personal communications with past participants indicate that active interaction on social media and private visits are still ongoing with involvement dating back to the initial travel participants of the program. Long-term follow-up will provide data on this subject.

#### International peer-to-peer teamwork

Limited reports are available on programs that connect students from different countries to each other [35,36]. While national and international student networking and travel programs do exist [37,38], typically they are not based on frameworks orchestrated by medical schools [2,30,36].

There is undeniable value to student-run programs via large organizations (i.e., IFMSA). Direct involvement in peer connection via medical schools and senior medical teaching faculty can be of value for quality control and integration into medical school curricula. As globalization will play a larger role in medical education, medical schools should take on an active role in student interactions.

#### Bidirectional student mobility

Student outbound mobility has long been regarded as synonymous with internationalization in medical education [6]. Eight-seven percent of US medical schools offer international programs for medical students [13,39] and almost 30% of medical students engage in a reported international activity during medical school [40]. Although there are other elements for IoME (i.e., internationalization at home and “glocal” programs [6,41,42]) student travel is still an attractive means to interest students in international and global healthcare issues.

Programs with multidirectional student exchanges are limited [28,43]. This program offered a bidirectional exchange involving traveling students who subsequently became the hosts for peers that visited them at their home institutions. These visits also boosted internationalization of the host campuses as an element of IoME.

#### International partnerships

International collaborations in medical education have been presented in various forms and directions [18,35,44,45,46,47,48]. The novelty of the program presented here is that twelve anatomy departments on four continents worked together to enhance medical and dental education for preclinical students and provided them with a platform for international networking, with the goal in mind of helping to create a generation of global citizen physicians and dentists. These types of faculty-driven collaborations in medical education have been reported but are still limited in scope [27]. This lack of reporting may lead to inefficiency, because departments seeking to establish new programs have no references to find best practices and will have to constantly “reinvent the wheel”. Anatomy to date has not been formally linked to GH education. Valuable learning objectives achieved through the anatomy cadaver dissection laboratory include teamwork, scientific thinking, ethics, and professionalism [49,50,51]. Despite this, traditional anatomy courses are often regarded as dated, costly, time consuming, and resource draining.

The majority of students in this program supported the idea of having anatomy as an anchor for the international exchanges. Due to the limited amount of time the students could spend with their peers, a significant number of groups decided not to focus on learning anatomy related topics from each other during their small group sessions. This demonstrates that students appreciated the networking opportunity initiated via the anatomy course. With the exception of reports generated by this group of authors there are no reports that link the discipline of anatomy to internationalization of medical education [2,30].

There are several advantages to choosing anatomy as an anchor for international activities. First, anatomy is a subject field that is taught universally in all medical and dental schools. Secondly, anatomy is typically taught very early in the preclinical phase of medical education. Many US medical schools offer GH experiences between years 1 and 2 of medical school [39]. Therefore, early linking of anatomy with IoME is in line with the concept of early GH exposure. Anatomy can serve as a vehicle to connect schools via collaboration on the faculty level. Because most anatomists are tightly linked with the basic sciences, internationalization efforts helps to attract students with research interests to international work and can open up areas of research that are not typically addressed in traditional research portfolios (e.g., research in the genetics of tropical diseases). Anatomy educators work very closely with students, have dedicated teaching time with them, and personal mentorships are established at an early stage. Through this mentorship the current program provided very individualized, custom-tailored international research laboratory placements for the students in phase 1.

### Content of student learning

#### Beyond anatomy

The content of this program differed from many international programs [12,22,23] because it mainly focused on acquiring knowledge pertaining to differences in healthcare systems, healthcare education, health ethics and law, public health, and not on clinical knowledge. The program was not meant to provide students with an in-depth study of Public Health, or intended as a replacement for a GH class. The peer-to-peer interaction rather was considered as inspiration and an enrichment, similar to additive courses described in the literature [52].

#### Intercultural Exchange

Cultural awareness is important for global leaders and should be part of the medical curriculum [53]. The cultural introduction within the large student conferences of this program helped the students to familiarize themselves with cultural differences. Unlike programs which focus on differences with LMIC, phase 1 elected to focus on countries that are of similar economic backgrounds, to ease young students into awareness of cultural differences in a more subtle way.

### Limitations

This program is primarily meant as an educational program. This paper presents qualitative research data (i.e., students’ subjective evaluation of what they have learned, liked, or appreciated). Long-term effects of these early international experiences on the student’s lives and careers will not be immediately available, and the authors are aware that there will be a lag time between the start of the program and potential longitudinal data, along with the challenges of keeping in contact with the participating students [54].

In the available literature there are not agreed upon learning objectives for GH programs, which will pose another challenge should one attempt to study learning objectives and outcomes for this program [12,20,23].

Lastly, as with any new emerging field in medicine, funding is limited until an official acknowledgement of the field is achieved. While some funds for students’ activities via institutional GH programs were secured, self-funding and the voluntary dedication of participating faculty are the norm.

Nevertheless, the program represented a substantial international student networking and exchange program, and reflected a new and additional role that the anatomist and the anatomy course can play in GH and the IoME.

The current report about an international partnership of anatomists in a new area of medical education – seemingly distant from pure anatomy teaching – can inspire future opportunities for collaboration in this regard.

## Conclusions

International collaboration among anatomy departments can lead to enhancement of student education by facilitating international experiences. This unique collaboration represented a new role for anatomy departments in GH and IoME – beyond the classic educational role of anatomy – and introduced a new purpose for international collaboration among anatomists.

## Additional File

The additional file for this article can be found as follows:

10.5334/aogh.2665.s1 Additional File. International Exchange Program Questionnaire 2018/19.

## References

[1] De Wit H, Hunter F, Howard L, Egron-Polak E. Internationalisation of Higher Education Brussels: European Parliament; 2015.

[2] Gölkel C, Wu A, Chiuzan C, Duong J, Bernd P, Kielstein H. Early internationalization of students in a German medical school in the former German Democratic Republic. Annals Of Anatomy = Anatomischer Anzeiger: Official Organ Of The Anatomische Gesellschaft; 2019 DOI: 10.1016/j.aanat.2019.03.004 30930197

[3] Knipper M, Baumann A, Hofstetter C, Korte R, Krawinkel M. Internationalizing Medical Education: The Special Track Curriculum ‘Global Health’ at Justus Liebig University Giessen. GMS Zeitschrift fur medizinische Ausbildung. 2015; 32(5): Doc52. 26604994 10.3205/zma000994 PMC4647159

[4] Majoor GD, Willemstein SC. Internationalization of medical education. Nederlands tijdschrift voor geneeskunde. 1996; 140(2): 100–102. 8569915

[5] Niemantsverdriet S, Majoor GD, Van Der Vleuten CPM, Scherpbier AJJA. Internationalization of medical education in the Netherlands: state of affairs. Medical Teacher. 2006; 28(2): 187–189. DOI: 10.1080/01421590500271225 16707304

[6] Stütz A, Green W, McAllister L, Eley D. Preparing Medical Graduates for an Interconnected World: Current Practices and Future Possibilities for Internationalizing the Medical Curriculum in Different Contexts. Journal of Studies in International Education. 2015; 19(1): 28–45. DOI: 10.1177/1028315314536991

[7] Koplan J, Bond TC, Merson M, Reddy KS, Rodriguez MH, Sewankambo NK. Towards a common definition of global health. Lancet. 2009; 373 DOI: 10.1016/S0140-6736(09)60332-9 19493564 PMC9905260

[8] Hanson L. Internationalising the Curriculum in Health In: Green W, Whitsed C (eds.), Critical Perspectives on Internationalising the Curriculum in Disciplines. 2015; 153–158. Springer DOI: 10.1007/978-94-6300-085-7_12

[9] Warner G. Internationalization Models and the Role of the University. International Education Magazine. 1992; 8(1): 21.

[10] Grudzen CR, Legome E. Loss of international medical experiences: Knowledge, attitudes and skills at risk. BMC Medical Education. 2007; 7: 47 DOI: 10.1186/1472-6920-7-47 18045481 PMC2242732

[11] Harden RM. International medical education and future directions: A global perspective. Acad Med. 2006; 81: S22–S29. DOI: 10.1097/01.ACM.0000243411.19573.58 17086041

[12] Battat R, Seidman G, Chadi N. Global health competencies and approaches in medical education: a literature review. BMC Medical Education. 2010; 10 DOI: 10.1186/1472-6920-10-94 21176226 PMC3019190

[13] Peluso MJ, Forrestel AK, Hafler JP, Rohrbaugh RM. Structured global health programs in U.S. medical schools: A web-based review of certificates, tracks, and concentrations. Acad Med. 2013; 88(1): 124–130. DOI: 10.1097/ACM.0b013e3182765768 23165271

[14] Kritz MM. Globalisation and Internationalisation of Tertiary Education: (http://www.un.org/en/development/desa/population/events/pdf/other/turin/P02_Kritz.pdf) http://wileyeditingservices.com/. INTERNATIONAL SYMPOSIUM ON INTERNATIONAL MIGRATION AND DEVELOPMENT Department of Economic and Social Affairs; 2006.

[15] ERASMUS. 2019; https://www.erasmusprogramme.com/post/what-is-the-erasmus-programme.

[16] DAAD. 2019; https://www.daad.de/deutschland/studienangebote/international-programmes/en/

[17] Ozturgut O, Cantu M, Pereira L, Krohn D. Effective strategies in internationalization of higher education in the United States. Vol 32014.

[18] Latifi R, Dasho E, Shatri Z, et al. Telemedicine as an Innovative Model for Rebuilding Medical Systems in Developing Countries Through Multipartnership Collaboration: The Case of Albania. Telemedicine & e-Health. 2015; 21(6): 503–509. DOI: 10.1089/tmj.2014.0138 25347524

[19] Sherer R, Dong H, Yunfeng Z, et al. Medical education reform in wuhan university, china: A preliminary report of an international collaboration. Teaching and Learning in Medicine. 2013; 25(2): 148–154. DOI: 10.1080/10401334.2013.770745 23530677

[20] Arthur MA, Battat R, Brewer TF. Teaching the basics: Core competencies in global health. Infectious Disease Clinics of North America. 2011; 25(2): 347–358. DOI: 10.1016/j.idc.2011.02.013 21628050 PMC7135705

[21] Holmes D, Zayas LE, Koyfman A. Student objectives and learning experiences in a global health elective. J Community Health. 2012; 37(5): 927–934. DOI: 10.1007/s10900-012-9547-y 22367606

[22] Izadnegahdar R, Correia S, Ohata B, et al. Global health in Canadian medical education: current practices and opportunities. Acad Med. 2008; 83(2): 192–198. DOI: 10.1097/ACM.0b013e31816095cd 18303368

[23] Khan OA, Guerrant R, Sanders J, et al. Global health education in U.S. medical schools. BMC Medical Education. 2013; 13(3). DOI: 10.1186/1472-6920-13-3 PMC3637494 23331630

[24] Mews C, Schuster S, Vajda C, et al. Cultural Competence and Global Health: Perspectives for Medical Education – Position paper of the GMA Committee on Cultural Competence and Global Health. GMS Journal for Medical Education. 2018; 35(3): Doc28. 30186938 10.3205/zma001174 PMC6120152

[25] Drain PK, Primack A, Hunt DD, Fawzi WW, Holmes KK, Gardner P. Global health in medical education: A call for more training and opportunities. Acad Med. 2007; 82(3): 226–230. DOI: 10.1097/ACM.0b013e3180305cf9 17327707

[26] Hayes JA, Ivanusic JJ, le Roux CM, et al. Collaborative development of anatomy workshops for medical and dental students in Cambodia. Anatomical Sciences Education. 2011; 4(5): 280–284. DOI: 10.1002/ase.238 21710643

[27] Strkalj G, Dayal M. Working together, sharing resources: An interuniversity collaboration to advance anatomy education. Anatomical Sciences Education. 2014; 7(6): 501–502. DOI: 10.1002/ase.1496 25288218

[28] Elharram M, Dinh T, Lalande A, Ge S, Gao S, Noel G. Global Health Values of a Multidirectional Near Peer Training Program in Surgery, Pathology, Anatomy, Research Methodology, and Medical Education for Haitian, Rwandan, and Canadian Medical Students. Annals of Global Health. 2017; 83(2): 274–280. DOI: 10.1016/j.aogh.2017.04.003 28619402

[29] Murray J. How Basic Science can Help to Inform Public Health and Improve Lives Globally. In: Foundation BaMG, ed2018.

[30] Wu A, Kielstein, H, Sakurai T, Noel G, Viranta-Kovanen S, Chien CL, Bernd P. Internationalization of Medical Education—Building a Program to Prepare Future Leaders in Healthcare. Medical Sciences Education; 2019 DOI: 10.1007/s40670-019-00695-4 PMC8368475 34457511

[31] Teichholtz S, Kreniske JS, Morrison Z. Teaching Corner: An Undergraduate Medical Education Program Comprehensively Integrating Global Health and Global Health Ethics as Core Curricula: Student Experiences of the Medical School for International Health in Israel. Journal of Bioethical Inquiry. 2015; 12(1): 51–55. DOI: 10.1007/s11673-014-9602-8 25630594

[32] Kalbarczyk A, Nagourney E, Martin NA, Chen V, Hansoti B. Are you ready? A systematic review of pre-departure resources for global health electives. BMC Medical Education. 2019; 19(1): 166 DOI: 10.1186/s12909-019-1586-y 31118015 PMC6532266

[33] Tillmanns RW, Ringwelski A, Kretschmann J, Spangler LD, Curry RH. The profession of medicine: A joint US-German collaborative project in medical education. Medical Teacher. 2007; 29(9): e269–275. DOI: 10.1080/01421590701551706 18158651

[34] Magarik J, Kavolus J, Louis R. An American medical student’s experience in global neurosurgery: Both in their infancy. World Neurosurgery. 2012; 77(1): 28–31. DOI: 10.1016/j.wneu.2010.05.039 22079820

[35] Finlayson AET, Baraco A, Cronin N, et al. An international, case-based, distance-learning collaboration between the UK and Somaliland using a real-time clinical education website. Journal of Telemedicine and Telecare. 2010; 16(4): 181–184. DOI: 10.1258/jtt.2010.004004 20511568

[36] Ambrose M, Murray L, Handoyo NE, Tunggal D, Cooling N. Learning global health: A pilot study of an online collaborative intercultural peer group activity involving medical students in Australia and Indonesia. BMC Medical Education. 2017; 17(1): 10 DOI: 10.1186/s12909-016-0851-6 28086875 PMC5237179

[37] IFMSA. International Federation of Medical Students’ Associations. 2019 https://ifmsa.org/.

[38] SGA. https://www.studentsgoabroad.com.

[39] McKinley DW, Williams SR, Norcini JJ, Anderson MB. International exchange programs and U.S. medical schools. Acad Med. 2008; 83(10 Suppl): S53–57. DOI: 10.1097/ACM.0b013e318183e351 18820502

[40] AAMC AoAMC. Medical School Graduation Questionnaire. 2017.

[41] Rowthorn V. Global/Local: What Does It Mean for Global Health Educators and How Do We Do It? Annals of Global Health. 2015; 81(5): 593–601. DOI: 10.1016/j.aogh.2015.12.001 27036715

[42] Kulkarni A, Francis ER, Clark T, Goodsmith N, Fein O. How we developed a locally focused Global Health Clinical Preceptorship at Weill Cornell Medical College. Medical Teacher. 2014; 36(7): 573–577. DOI: 10.3109/0142159X.2014.886764 24597684 PMC8052984

[43] Rohrbaugh R, Kellett A, Peluso MJ. Bidirectional Exchanges of Medical Students Between Institutional Partners in Global Health Clinical Education Programs: Putting Ethical Principles into Practice. Annals of Global Health. 2016; 82(5): 659–664. DOI: 10.1016/j.aogh.2016.04.671 28283116

[44] Anderson F, Donkor P, de Vries R, et al. Creating a charter of collaboration for international university partnerships: the Elmina Declaration for Human Resources for Health. Acad Med. 2014; 89(8): 1125–1132. DOI: 10.1097/ACM.0000000000000384 24918757

[45] Geffen L, Cheng B, Field M, Zhao S, Walters T, Yang L. Medical school accreditation in China: A Sino-Australian collaboration. Medical Teacher. 2014; 36(11): 973–977. DOI: 10.3109/0142159X.2014.917286 25072750

[46] Jotkowitz AB, Gaaserud A, Gidron Y, Urkin J, Margolis CZ, Henkin Y. Evaluation of student attitudes and knowledge in a new program in international health and medicine. Medical Teacher. 2004; 26(6): 574–576. DOI: 10.1080/01421590410001711571 15763839

[47] Mayo A. Improving medical education in Kenya: An international collaboration. Journal of the Medical Library Association: JMLA. 2014; 102(2): 96–100. DOI: 10.3163/1536-5050.102.2.007 24860265 PMC3988781

[48] Risley B, Foley RP, Nooman ZM, Richards RW, Ezzat E, Maklady F. A collaboration between two innovative medical education programmes in Egypt and the United States. Medical Education. 1989; 23(4): 333–338. DOI: 10.1111/j.1365-2923.1989.tb01558.x 2770575

[49] Lachman N, Pawlina W. Integrating professionalism in early medical education: The theory and application of reflective practice in the anatomy curriculum. Clin Anat. 2006; 19 DOI: 10.1002/ca.20344 16683241

[50] Pawlina W, Drake RL. Driving effective communication through anatomy. Anatomical Sciences Education. 2008; 1(2): 49 DOI: 10.1002/ase.17 19177380

[51] Drake RL, McBride JM, Pawlina W. An update on the status of anatomical sciences education in United States medical schools. Anatomical Sciences Education. 2014; 7(4): 321–325. DOI: 10.1002/ase.1468 24895314

[52] Eaton DM, Redmond A, Bax N. Training healthcare professionals for the future: internationalism and effective inclusion of global health training. Medical Teacher. 2011; 33(7): 562–569. DOI: 10.3109/0142159X.2011.578470 21696283

[53] Dogra N, Reitmanova S, Carter-Pokras O. Teaching cultural diversity: current status in U.K., U.S., and Canadian medical schools. J Gen Intern Med. 2010; 25(Suppl 2): S164–168. DOI: 10.1007/s11606-009-1202-7 20352513 PMC2847109

[54] Umoren RA, Gardner A, Stone GS, et al. Career choices and global health engagement: 24-year follow-up of U.S. participants in the Indiana University-Moi University elective. Healthcare (Amsterdam, Netherlands). 2015; 3(4): 185–189. DOI: 10.1016/j.hjdsi.2015.10.001 26699341

