# Supplementary material for: An International Partnership of 12 Anatomy Departments – Improving Global Health through Internationalization of Medical Education
Source: Ann Glob Health. 2020 Mar 6;86(1):27. doi: 10.5334/aogh.2665 (PMC7059426; doi:10.5334/aogh.2665)
Supplement: Additional File. — International Exchange Program Questionnaire 2018/19. [file agh-86-1-2665-s1.pdf]

AN INTERNATIONAL PARTNERSHIP OF 12 ANATOMY DEPARTMENTS – IMPROVING GLOBAL HEALTH THROUGH INTERNATIONALIZATION OF MEDICAL EDUCATION

International Exchange Program Questionnaire 2018/19

General - I am a

- ○ Medical student
- ○ Dental student

General - Indicate your age group

- ○ under 20
- ○ 20-25
- ○ over 25

General - I am

- ○ Male
- ○ Female
- ○ Prefer not to disclose

General - Language

- ◯ My native language is English
- ◯ My native language is ____

SMALL GROUP- **Before** the small group sessions... (move sliders, 1= none 10= a lot)

	0 1 2 3 4 5 6 7 8 9 10
I had prior knowledge about the health education system in the partner countries	
I had prior knowledge about the health delivery system in the partner countries	
I had prior knowledge about the public health challenges in my own country	
I had prior knowledge about the public health challenges in the partner countries	
I had prior knowledge about health ethics issues in my own country	
I had prior knowledge about health ethics issues in the partner countries	
I had prior knowledge about the culture in the partner countries	
I knew people in the partner countries prior to the group sessions	

SMALL GROUP EXCHANGE. On a scale of 1-10, how much you liked or disliked this experience (0= dislike, 10= liked it very much)

	0 1 2 3 4 5 6 7 8 9 10
I liked/disliked the small group student interactions	

SMALL GROUP EXCHANGE -Time spent/duration of sessions

- ○ We spoke/interacted for one hour each time
- ○ We spoke/interacted for about 2 hours each time
- ○ We spoke/interacted for more than 2 hours each time

We spent more time with each other because (check all that applies)

- ☐ The scheduled time did not allow us to cover everything we wanted to talk about
- ☐ We lost track of time because it was very interesting
- ☐ We liked our peers and became friends

SMALL GROUP EXCHANGE - Number of group meetings per session

- ○ We only spoke on the scheduled sessions (session 1-4)
- ○ We added one more group session
- ○ We added more than 2 additional sessions

SMALL GROUP EXCHANGE - Scheduling

- ○ It was difficult to schedule a convenient/mutual time to speak, and we had to skip sessions or combine them
- ○ We managed to find time but it was often challenging
- ○ It was not a problem for us to schedule a convenient/mutual time to speak

---

SMALL GROUP EXCHANGE - ANATOMY - Do you think you learned something new from your international peers about their respective Anatomy courses (and their experience of Anatomy); that is different from your own experience?

- ○ No
- ○ Yes

If so - what did you learn that was different? List as many examples as you can give.

____

SMALL GROUP EXCHANGE - ANATOMY - Do you think you learned something about Anatomy itself from your peers?

- ○ Yes
- ○ Not sure
- ○ No

SMALL GROUP EXCHANGE - ANATOMY-Is there anything you would like to comment on, regarding the Anatomy session? Write all that you think will help improve this program going forward in regard to Anatomy.

____

SMALL GROUP - **After** the sessions. Indicate what you learned (1 = none; 10 = a lot)

0	1	2	3	4	5	6	7	8	9	10

I learned about the healthcare education system in the partner countries	
I learned about the healthcare delivery system in my own country	
I learned about the healthcare delivery system in the partner countries	
I learned about public health challenges in my own country	
I learned about public health challenges in the partner countries	
I learned about health ethics in my own country	
I learned about health ethics in the partner countries.	

SMALL GROUP EXCHANGE - Please note the most interesting thing(s) that you learned.

____

SMALL GROUP EXCHANGE - Was the selection of discussion topics appropriate?

- ○ No - I would have liked different topics
- ○ I have no opinion
- ○ Yes - the selection of discussion topics was appropriate

What topics would you like to have included, that are relevant for your knowledge/learning? List as many as you like in the order of preference.

____

SMALL GROUP EXCHANGE - **After** the sessions. General learning objectives (1 = none, 10 = a lot)

	0	1	2	3	4	5	6	7	8	9	10

I learned how to work with international peers/colleagues	
It enhanced my ability to take ownership and leadership	
I learned to pay attention to cultural differences	
I practiced how to act as a medical professional	
I feel I am now more open to other cultures and ideas	
I am more appreciative of what I have in my own institution and country after I spoke with my peers	
I have a better understanding of other systems and languages	
I feel I am more patient and empathic towards non-English speaking peers	
I improved my foreign language skills	

LARGE GROUP VIRTUAL CONFERENCE - **Prior** to the conference (1 = none, 10 = a lot)

0 1 2 3 4 5 6 7 8 9 10

I have presented internationally before	
I have good public speaking/presentation skills	
I have heard non-native English speakers present	

LARGE GROUP VIRTUAL CONFERENCE - What was your overall experience of the large group meeting?

- ◯ I liked it
- ◯ I am not sure if I liked it
- ◯ I did not like it

If you did not like it - what suggestions do you have for improving the virtual conference? List as many as you like.

____

LARGE GROUP VIRTUAL CONFERENCE - Part I - Learning from the intercultural exchange (1 = I do not agree; 10= I agree)

	0 1 2 3 4 5 6 7 8 9 10
I enjoyed learning about the other countries' culture	
It was helpful to have additional information about the partner counties	
This information is important for me - to help decide whether I would like to visit the country	
It has helped me to become more open minded/a global citizen	

LARGE GROUP VIRTUAL CONFERENCE - Part II - Learning about healthcare education systems (1= none; 10= a lot)

	0 1 2 3 4 5 6 7 8 9 10
After the conference - my knowledge about other healthcare education systems has improved	

LARGE GROUP VIRTUAL CONFERENCE - Part II - Learning about Public Health and Health Ethics (1= I do not agree; 10= I agree)

	0 1 2 3 4 5 6 7 8 9 10
After the conference - my knowledge about international Public Health and Health Ethics issues has increased	
After the conference - I am more aware/understanding of these topics	
After the conference - I feel better prepared for addressing Public Health issues	

LARGE GROUP VIRTUAL CONFERENCE - **After** the conference. General learning objectives (1 = I do not agree; 10 = I agree very much)

	0 1 2 3 4 5 6 7 8 9 10
My public speaking skills have improved	
I learned how to listen to other speakers - most of them were not native English-speaking students	
It helped me to bond with my international peers	
It was a good exercise and preparation for future international collaboration	
It was a good way to present our work	

LARGE GROUP VIRTUAL CONFERENCES - TIME

- ○ It was too short
- ○ It was just right
- ○ It was too long

LARGE GROUP VIRTUAL CONFERENCES -General comments and suggestions for future conferences

____

GENERAL - The program interactions contributed to my understanding of global health.

- ◯ No
- ◯ Maybe
- ◯ Yes

GENERAL - This interaction has inspired me to learn more about the country (culture and medical system) of my peers.

- ◯ No, I do not agree
- ◯ I have no opinion
- ◯ Yes, I agree

GENERAL - Do you think this type of interaction should be part of the medical school curriculum?

- ◯ No
- ◯ Maybe
- ◯ Yes

Why do you think this should NOT be part of the medical school curriculum? List one or more reasons.

____

Why do you think this should be part of the curriculum? List one or more reasons.

____

GENERAL- If this study was part of an official program would you like it to be continued in the clinical years?

- ○ No
- ○ Maybe
- ○ Yes

GENERAL - Do you think this international peer-to-peer interaction should be started in the Anatomy course?

- ○ No- I prefer it to be part of another course/class
- ○ I have no opinion
- ○ Yes- Anatomy is a good course to start this international experience

If you think this peer-to-peer interaction should NOT be started in the Anatomy course please suggest when this interaction should occur (i.e., second, third, final year)?

GENERAL - I will keep in contact with my international peers.

- No, unlikely
- Maybe
- Yes, most likely

GENERAL - What changes do you recommend for this program?

FUTURE PLANS - I am interested in a continuation of the international small group work in the clinical years

- Yes
- Maybe
- No

FUTURE PLANS - I am interested in keeping in contact with my current group

- Yes
- Maybe
- No - prefer to be matched with other students

PRE TRAVEL

Preparation - Is it helpful to have a Skype partner in the host country that you know before, to help with travel preparations etc.?

- ○ Yes
- ○ Not sure
- ○ No

Research experience - Rate your overall previous (basic) sciences research experience **prior** to your visit (1= low, 5= very good)

	0	1	2	3	4	5
I rate my previous research experience as...						

I have had experience in the following research techniques (e.g., data analysis, flow cytometry, literature searches, etc.; list as many as you like)

____

Research experience details **before** the internship - I have had knowledge about these techniques prior to my visit. Please rate your skills prior to the visit (0=none, 5= I knew about this very well)

0	1	2	3	4	5

sterile techniques	
cell culture	
flow cytometry	
PCR	
data collection	
statistics	
data analysis	
data presentation	
project design	
working with research animals	
writing grants	
writing research protocols	
working with human subjects in research	

Research Experience - Do you think it will be helpful for your career to have worked in a research lab?

- ○ Yes
- ○ Maybe
- ○ No

Research Experience - Why do you think it is important for your career to have gone abroad?

____

Cultural learning - **Prior** to the visit - what do you know about the culture of your host country? List all you know (including stereotypes). Please write "zero" if you do not know.

____

Overall program evaluation - Do you think this visit is an appropriate continuation of the Anatomy Skyping project?

- ◯ Yes
- ◯ Maybe
- ◯ No

Overall program evaluation - Do you think you are well prepared for this visit?

- ◯ Definitely yes
- ◯ Probably
- ◯ No, I do not feel prepared

Overall program evaluation - Do you think you would have liked to have other choices of countries that you could have visited at your current stage of education?

- ◯ The current choices are fine
- ◯ Not sure
- ◯ I would have liked more choices

POST TRAVEL

Q11 Research Experience - **post** visit. Rate your overall (basic) sciences experience **after** the visit. (1= low, 5= very good)

0	1	2	3	4	5

I rate my research skills after the visit as...	

Q12 I now have research experience in ... (list as many as you can)

____

Q13 Research Experience - **post** visit. How would you rate the experience in your host lab/host research group? Move the sliders to the right as appropriate (left= do not agree -> right =strongly agree).

	0 1 2 3 4 5
My mentor was very helpful and provided good feedback	
My project and my role were clear to me	
The lab was well equipped	
There were no/little language barriers	
I was welcomed on to the team	
My mentor spent time with me, to teach me and to answer my questions	

Q14 Research Experience - **post** visit. How do you rate your skills after the visit? Check as many things as you think you have learned in the lab (0= I did not learn this technique, 10= I have learned a lot about this technique)

0 1 2 3 4 5

sterile techniques	
cell culture	
flow cytometry	
PCR	
data collection	
statistics	
data analysis	
data presentation	
project design	
working with research animals	
writing grants	
writing research protocols	
working with human subjects in research	

Research experience - **post** visit. In your own words what techniques have you learned in addition to the ones you knew before?

Research Experience - Do you think it was helpful for your career to have worked in a research lab?

- ◯ Yes
- ◯ Maybe
- ◯ No

Overall program evaluation - What are the three most important things you learned during the trip?

____

Overall program evaluation - What did you like most about your visit?

____

After the program - I will remain in contact with my international peers.

- ◯ Yes
- ◯ Maybe
- ◯ No

Overall program evaluation - Do you think you were well prepared for this visit?

- ◯ Definitely yes
- ◯ Probably
- ◯ No, I did not feel prepared

Cultural learning - **after** the program (1= do not agree, 10= strongly agree)

	0 10 20 30 40 50 60 70 80 90 100
I feel I have a better understanding of another culture	
I feel more tolerant towards another culture	
I feel more empathic towards another culture	
I feel more appreciative of what I have at home	
I feel my language skills improved	

General learning - This experience (move sliders to right on all that apply)

not much somewhat very much

0 1 2 3 4 5

...enhanced my foreign language skills	
...influenced my future career plans	
...influenced change in my life style	
...changed my world-view	
...increased my understanding of my home country	
...increased my self- confidence	
...increased my appreciation of what I have at home	
...increased my tolerance of other cultures and countries	
...prompted me to consider working abroad	
...increased my ability to adapt to new and different situations	
...changed my values, priorities, and goals	
...influenced my professional and academic direction	
...increased my ability to work and collaborate internationally in the future	
...enhanced my cultural knowledge	
...enhanced my networking pool, and increased my ability to network	

Overall program evaluation - What are the three most important things you learned during the trip?

____

Overall program evaluation - What did you like most about your visit?

____

Overall program evaluation - Do you think this visit is an appropriate continuation of the Anatomy Skyping project?

- ◯ Yes
- ◯ Maybe
- ◯ No

After the program - I am interested in more visits to other countries in my clinical years for clinical rotations and more scholarly/research work.

- ◯ Yes
- ◯ Maybe
- ◯ No

After the program - From what you can tell now - do you think this visit and the Skyping experience will have an impact on your career or career choice?

- ◯ Yes
- ◯ Most likely
- ◯ Undecided, too early to tell
- ◯ Probably not
- ◯ No

After the program - I will remain in contact with my international peers.

- ○ Yes
- ○ Maybe
- ○ No
